# Enhancement of image quality and imaging depth with Airy light-sheet microscopy in cleared and non-cleared neural tissue

**DOI:** 10.1364/BOE.7.004021

**Published:** 2016-09-14

**Authors:** Jonathan Nylk, Kaley McCluskey, Sanya Aggarwal, Javier A. Tello, Kishan Dholakia

**Affiliations:** 1 SUPA, School of Physics and Astronomy, University of St. Andrews, St. Andrews, KY16 9SS, UK; 2 School of Medicine, University of St. Andrews, St. Andrews, KY16 9TF, UK

**Keywords:** (070.0070) Fourier optics and signal processing, (100.2960) Image analysis, (100.2980) Image enhancement, (170.3880) Medical and biological imaging, (180.0180) Microscopy, (180.2520) Fluorescence microscopy

## Abstract

We have investigated the effect of Airy illumination on the image quality and depth penetration of digitally scanned light-sheet microscopy in turbid neural tissue. We used Fourier analysis of images acquired using Gaussian and Airy light-sheets to assess their respective image quality versus penetration into the tissue. We observed a three-fold average improvement in image quality at 50 μm depth with the Airy light-sheet. We also used optical clearing to tune the scattering properties of the tissue and found that the improvement when using an Airy light-sheet is greater in the presence of stronger sample-induced aberrations. Finally, we used homogeneous resolution probes in these tissues to quantify absolute depth penetration in cleared samples with each beam type. The Airy light-sheet method extended depth penetration by 30% compared to a Gaussian light-sheet.

## 1. Introduction

Light-sheet microscopy (LSM) is an emergent fluorescence microscopy technique already showing great promise in biomedical research. LSM enables rapid, high-contrast, optically sectioned visualization of large three-dimensional samples with minimal photo-damage and is therefore ideally suited for imaging studies in developmental biology [1] and neuroscience [2,3].

Mammalian brains are extremely complex systems consisting of billions of neurons, of which hundreds of genetically distinct cell types form unique connectivity patterns. The benefits of LSM have become apparent in systems neuroscience by providing researchers with the ability to extract anatomical-projection information (often spanning multiple brain areas) and activity patterns from multiple neurons with high temporal and spatial resolution. While imaging fairly transparent specimens such as a zebrafish brain is achievable using current LSM systems [2], the ability to image larger less transparent turbid specimens, and in the presence of strong aberrations is desirable to advance neuroscience research. Few studies have investigated the performance of LSM in highly turbid media, and these are largely theoretical works (for example, [4,5]).

The natural divergence of a Gaussian beam limits the optical sectioning ability of traditional LSM. If a large field-of-view (FOV) is required, a broad light-sheet must be used, while a narrow light-sheet can only be achieved over a small FOV. Use of propagation-invariant beam types in LSM, most notably Bessel [6–8] and Airy beams [9, 10], has extended the FOV of high-resolution, single-photon excitation LSM, as these beam types overcome diffraction and can maintain a constant beam profile over longer longitudinal distances than a Gaussian beam. The lower peak intensity used in Airy LSM further reduces photodamage in the sample [9].

Bessel beam-based LSM has been shown to offer some improvements over Gaussian LSM in terms of depth penetration, which has been attributed to self-healing [6, 11], the ability to recompose the transverse beam profile after propagation through an obstruction. This property has been extensively studied in both Bessel beams [12–14] and Airy beams [14–19], but all previous studies have only considered amplitude based obstructions whereas biological tissue is an ensemble of complex (amplitude and phase) objects. Recently, alternative mechanisms for the enhancement of Bessel beam-based imaging modalities have been proposed [20], which warrants further fundamental investigation. However, studies of a more applied nature are of equal merit. As the Airy beam exhibits similar propagation-invariant behaviour, the Airy beam is expected to give similar improvements to the Bessel beam in turbid media, however there is currently little investigation of Airy beam propagation in turbid biological tissue. The development of Airy-beam LSM techniques suitable for biomedical applications [9,10] warrants an assessment of the performance of the Airy LSM approach at depth in real neural tissue.

In this paper, we provide a quantitative comparison of Gaussian LSM (GLSM) and Airy LSM (ALSM) in mouse brain tissue. Key morphological features of fluorescently labelled neurons were easier to recognize in the ALSM rendering, especially at greater tissue depth. To quantify these results, we use Fourier analysis to develop an image quality metric for comparing imaging methods and find that ALSM gives a three-fold improvement at a depth of 50 μm into the tissue. The origin of this enhancement is investigated using optical clearing [21] to vary the scattering properties and aberrations of the tissue. Finally, homogeneous resolution probes are embedded in mouse brain tissue to quantify the maximum imaging depth with each beam type.

## 2. Methods

### 2.1. Airy light-sheet microscope

The ALSM is described in detail elsewhere [9]. In brief, a laser (Laser Quantum Finesse 5W, 532nm) was expanded to overfill a spatial light modulator (SLM; Hamamatsu LCOS X10468-04) programmed to display the appropriate phase mask for either Gaussian or Airy illumination. For a Gaussian light-sheet, the phase profile across the pupil is uniform, for an Airy light-sheet the phase profile is described by *P*(*u*, *v*) = exp (2*πiα*[*u*^3^ + *v*^3^]), where *u* and *v* are normalised pupil coordinates corresponding to the *z*– and *y*–axes respectively and *α* allows the propagation-invariance of the Airy beam to be tuned [9]. The SLM was imaged onto an acousto-optic deflector (AOD; Neos AOBD 45035-3), which generated a light-sheet by digital scanning, and then imaged onto the back aperture of the illumination objective (Nikon CFI Apo 40×/0.80 DIC, w.d. = 3.5mm, water immersion). The numerical aperture (NA) of the illumination was restricted to 0.42 holographically.

Fluorescence was collected through a second, identical objective with NA restricted to 0.4 and imaged onto an sCMOS camera (Hamamatsu, Orca Flash 4.0). Software for system control and data acquisition was written in-house and implemented in LabVIEW. Software for image processing and deconvolution was written in-house in MATLAB.

**Fig. 1:**
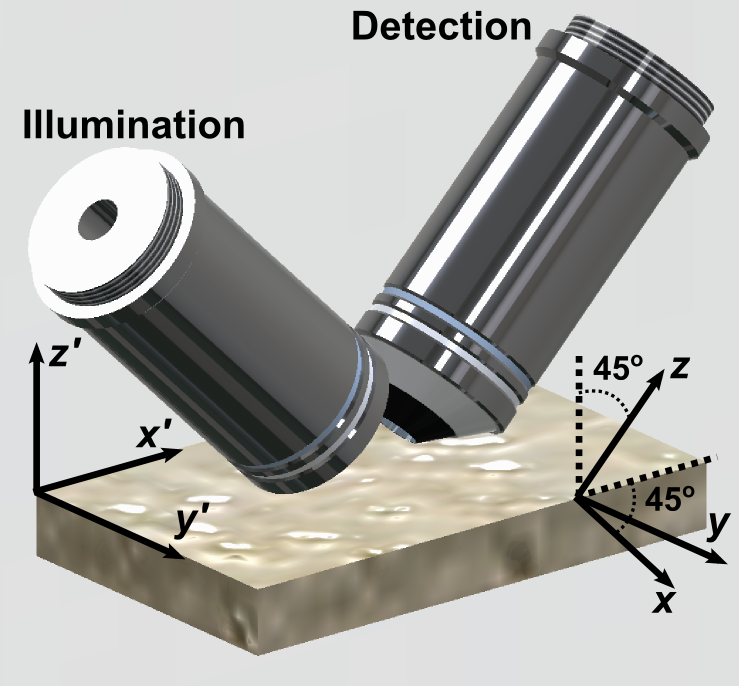
Schematic of the light-sheet microscope objectives, oriented 45° to the vertical, and sample slide. Primed coordinates: tissue reference frame. Unprimed coordinates: microscope reference frame. *y* = *y′*, while *x* and *z* are tilted 45° relative to *x′* and *z′* such that the focal plane of the illumination objective (left) is parallel to *x* − *y*, and *z* is parallel to the optical axis of the detection objective.

The microscope is configured in a “dual-inverted” geometry (Fig. 1). Throughout this paper, we refer to the laboratory and tissue reference frame with primed coordinates, *x′*, *y′*, and *z′*, where *z′* = 0 defines the top surface of the tissue section. The microscope reference frame is defined by unprimed coordinates, *x*, *y*, and *z*, where *z* is the optic axis of the detection objective lens and light-sheet propagation is in the +*x* direction.

Images of tissue sections were acquired using both GLSM and ALSM (Airy parameter: *α* = 7 [9]) modalities. Both modalities were used to image the same regions of the tissue to enable direct comparison. Given the system parameters, GLSM was expected give 800nm isotropic resolution over a FOV 16 μm wide, with axial resolution rapidly decaying outwith this FOV, ALSM was expected to give 800nm isotropic resolution over a FOV 340 μm wide [9]. Z-stacks were acquired over an axial distance of 200 μm with z-plane spacing of 400nm. The illumination power was kept constant for all experiments at 240μW. All ALSM datasets were deconvolved as described by Vettenburg *et al* [9]. Deconvolution was not performed on GLSM datasets, as this is not a strict requirement for the technique and introduces strong artefacts into regions of the image at the edge of, and outwith, the high-resolution FOV.

### 2.2. Mouse tissue preparation

Animal experiments were reviewed and approved by the University of St Andrews Animal Ethics and Welfare Committee under Dr Tello’s Home Office Project License 70/7924. A Cre recombinase (Cre)-dependent adenoassociated virus vector was used to target expression of mCherry (a monomeric red fluorescent protein) to hypothalamic *Kiss1*^+^ neurons in *Kiss1-creGFP* mice. All breeding and husbandry was performed at the University of St. Andrews, St. Mary’s Animal Unit. Mice heterozygous for the *Kiss1-creGFP* locus were obtained by breeding heterozygous *Kiss1*^tm1.1(cre/EGFP)Stei^/J mice. Subjects were weaned at 21 days and housed in same-sex groups, under regular light-dark cycles (12h light, 12h dark) with food and water available *ad libitum*.

#### Preparation of virally transduced tissue

Adult female heterozygous Kiss1-creGFP mice were anaesthetised with isofluorane, and viral particles (AAV; AAV1/2-Ef1a-DIO-mCherry-wPRE; 1.75*x*10^11^gc/ml; prepared in-house as described by McClure *et al* [22]) were stereotaxically injected bilaterally into the hypothalamic arcurate nucleus (coordinates: AP −1.6, ML ±0.3, DV −5.9) using a pulled glass pipette at a volume of 400nL/side, at a rate of 75nL/min using pressure injection. After surgery, mice were returned to their cages for 3 weeks to allow for viral vector activation.

#### Tissue preparation

Animals were deeply anaesthetised with an overdose of sodium pentobarbital (100mg/kg) and transcardially perfused with 0.1M PBS (pH 7.4) followed by 4% PFA in PBS (pH 7.4). Brains were removed from the skull and post-fixed overnight in 4% PFA in PBS and subsequently cryopreserved in 30% sucrose in 0.1M PBS. The brains were sectioned using a Compresstome vibratome (Precisionary Instruments VF-300) at a thickness of 400 μm.

#### Preparation of beads-injected tissue

Adult female wild type mice were anaesthetised as described previously. Fluorescent beads (Duke Scientific R600, 600nm diameter polystyrene, red fluorescence), diluted 1:50 in PBS, were stereotaxically injected with a volume of 500nL/side bilaterally into the arcuate nucleus as described previously. Mice were culled 2h following bead injection and post-fixed as described above. After clearing, the density of beads was significantly reduced and a further injection was performed on the fixed tissue.

#### Optical clearing

Tissue sections were optically cleared using TDE as described by Constantini *et al* [21]. The cleared tissue sections were embedded in 1% LMP agarose gel made with 47% TDE/PBS buffer and immersed in 47% TDE/PBS during imaging. Non-cleared tissue was embedded in 1% LMP agarose gel made with 0.1M PBS buffer and immersed in 0.1M PBS during imaging.

Imaging in the 47% TDE/PBS buffer is expected to introduce spherical aberration, as the refractive index is *n_TDE_* = 1.42 ± 0.01 [21], higher than the refractive index the objective lenses are designed for, *n* = 1.33. To understand the effects of this spherical aberration, samples of 600nm diameter red fluorescent polystyrene beads (Duke Scientific; polydispersity ±50nm) were embedded in 1% LMP agarose gel made with 0.1M PBS or 47% TDE/PBS buffers, immersed in the appropriate imaging buffer as described above, and imaged with both GLSM and ALSM modalities. A spot-finding algorithm (see Section 3.2) was used to identify isolated beads and determine their full width at half maximum (FWHM) along their lateral dimensions. The results are shown in Table 1. The results indicate that diffraction limited performance is achieved in PBS buffer as expected, and that the refractive index mismatch when imaging in TDE buffer reduces the resolution by between 20 − 30%. These datasets can be accessed at [23].

**Table 1:** FWHM lateral measurements of fluorescent resolution markers in 0.1M PBS and 47% TDE/PBS buffers. Sample size indicates number of beads detected by spot finding algorithm.

**Beam Type**	**Buffer Medium**	***x***–**FWHM (**μ**m)**	***y***–**FWHM (**μ**m)**	**Sample Size**

Gaussian	PBS	0.83 ± 0.05	0.77 ± 0.04	50
	TDE	1.1 ± 0.1	1.0 ± 0.1	129

Airy	PBS	0.79 ± 0.02	0.83 ± 0.06	48
	TDE	1.1 ± 0.2	1.0 ± 0.1	124

## 3. Results and discussion

### 3.1. Fourier analysis of virally transduced fluorescent tissue sections

**Fig. 2:**
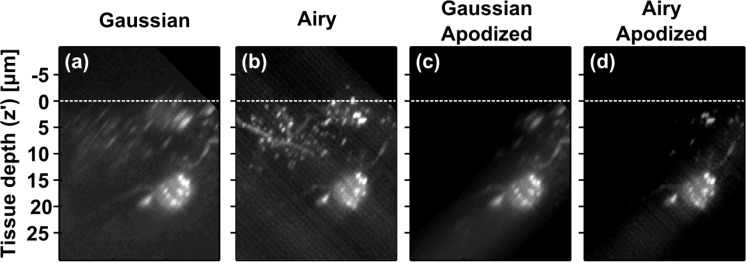
(a,b) Maximum intensity projections (*x′* − *z′* view) of fluorescent neurons in 400 μm thick, non-cleared mouse brain tissue section acquired using GLSM (a) and ALSM (b). Dashed white lines indicate tissue surface. (c,d) Apodized versions of (a,b) showing only the region around Gaussian beam focus which is used for analysis. This dataset can be accessed at [23].

Biological features within tissue typically exhibit structure across multiple length scales (Fig. 2(a,b)). This non-uniformity poses a challenge to assessing image quality by measurements in real-space. One alternative is to analyse the image in the spatial frequency domain. We developed a metric for image quality based on the spectral magnitude of the image within a given spectral window, and investigated this as a function of tissue depth. The spectral magnitude within the *n^th^* spectral window of an image of the *x′* − *y′* plane, *S_n_*(*z′*), is given by:

(1) $$S_{n}(z^{\prime })=\frac{\int_{k_{r,n-1}}^{k_{r,n}}\int_{0}^{2\pi }\left|\tilde{I}(k_{r},k_{\theta };z^{\prime })\right|k_{r}dk_{r}dk_{\theta }}{\int_{k_{r,n-1}}^{k_{r,n}}\int_{0}^{2\pi }k_{r}dk_{r}dk_{\theta }}$$

where *Ĩ*(*k_r_*, *k_θ_*; *z′*) is the Fourier transform of the image plane *I*(*x′*, *y′*; *z′*) in cylindrical coordinates and *k_r,n_* is the radial spatial frequency separating spectral windows in 10% increments of the diffraction limit:

(2) $$k_{r,n}=\frac{n}{10}\frac{2\text{NA}}{\lambda }$$

The enhancement factor within a given spectral window is then given by the ratio between Airy and Gaussian imaging modalities:

(3) $${\mathrm{EF}}_{n}(z^{\prime })=\frac{S_{n}{\left(z^{\prime }\right)}_{\mathrm{Airy}}}{S_{n}{\left(z^{\prime }\right)}_{\mathrm{Gaussian}}}$$

While the spectral magnitudes, *S_n_*, and enhancement factors, *EF_n_*, can, in principle, be analyzed along any axis, some directions will be more insightful than others. Analysis in the microscope coordinates, *x* or *z*, will show behaviour in the illumination or detection pathways separately; however, this is dependent on the orientation of the tissue. Following the convention set out by Glaser, Wang, and Liu [4], we perform this analysis along the depth axis of the tissue slice, *z′*. This metric concisely encapsulates the performance of both the illumination and detection imaging sub-systems and is applicable for all areas imaged within the tissue. It is interesting to note, nevertheless, that for any point *z′* deep in the tissue, the light-sheet has propagated a distance $\left|\sqrt{2}z^{\prime }\right|$ along the *x*-axis prior to reaching this location.

Figure 2(a,b) shows ALSM (b) and GLSM (a) images of labelled neurons in non-cleared mouse brain tissue. Qualitatively, Fig. 2(a,b) shows that ALSM gives a better quality image from a single imaging run. This improvement is due to an extended high-resolution field-of-view (FOV) and is covered in detail by Vettenburg *et al* [9]. Arguably, however, the qualitative comparison is unfair, as it compares regions of the image outwith the Gaussian light sheet’s FOV to regions within the Airy FOV. The inequity cannot be fixed by simply using a Gaussian light sheet with the same FOV as the ALSM system, as it would have a much lower maximum axial resolution (approx. 3.5 μm), again rendering the comparison unfair. Therefore, we have chosen Airy and Gaussian light sheet profiles with comparable best-performance axial resolution (800 nm) at the cost of differing fields-of-view, but restricted our numerical analysis to the region of the image where both light sheets can potentially achieve best performance.

**Fig. 3:**
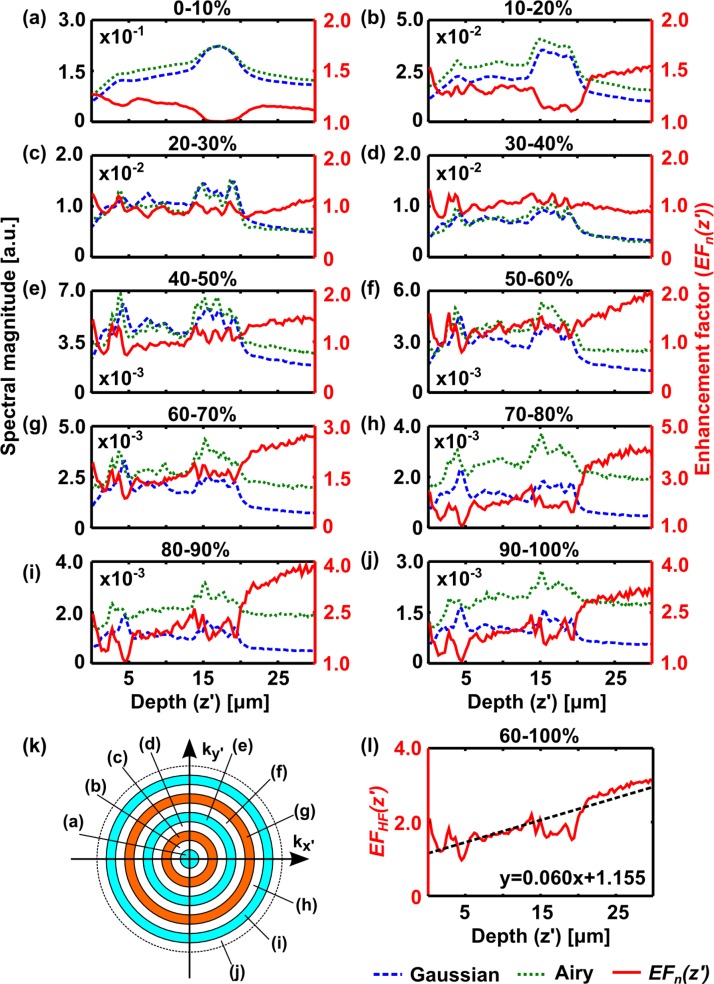
Explanation of Fourier analysis method. (a–j) Plots of normalised spectral magnitude, *S_n_* (*z′*), versus tissue depth for datasets shown in Fig. 2, acquired with Gaussian (dashed blue) and Airy (dotted green) light-sheet illumination within selected spectral windows, (a) 0 – 10% 2NA/*λ* – (j) 90 – 100% 2NA/*λ*. The enhancement factor, *EF_n_* (*z′*), (red; second axis) is also plotted. (k) Illustration of spectral windows used to segment the data for plots (a–j). The dashed circle corresponds to a spatial frequency of 2NA/*λ*. The annular regions each cover 10% of this frequency range. (l) shows the average enhancement factor over high-frequency spectral bands, *EF_HF_* (*z′*), (60 – 100% 2NA/*λ*). Dashed line in (l) shows a linear fit to the data, equation shown on plot.

To accurately compare best performance of each illumination type, the image was first apodized (Fig. 2(c,d)) so the analysis was only performed on the region around the Gaussian focus, ensuring only the region of the images where both imaging techniques give comparable resolution is analyzed. Specifically, the volume is apodized with a Gaussian function with 8 μm half-width along the *x*-axis, centred at the focus of the Gaussian light sheet. *S_n_*(*z′*) (Gaussian: dashed blue lines, Airy: dotted green lines; first axis) and *EF_n_*(*z′*) (solid red lines; second axis) is then determined (Fig. 3(a–j)). As this analysis is terminated at the spatial frequency corresponding to the diffraction limit, it is robust to single-pixel noise fluctuations.

Figure 3(a–j) show that for low spatial frequencies, both techniques have similar *S_n_*(*z′*). This indicates that both Gaussian and Airy acquisitions have similar intensity and are directly comparable, a good check to make for comparison as both images were acquired with equal illumination power. At high spatial frequencies, however, the enhancement factor steadily increases with increasing depth into the tissue, showing a clear improvement in relative image quality and indicating that ALSM is more resistant to sample-induced aberrations than GLSM.

An average enhancement factor, *EF_HF_* (*z′*), was taken over the high-frequency spectral bands between 60% and 100% of 2NA/*λ* (Fig. 3(l)) and fitted with a linear function in *z′*. The example shown in Fig. 3 yields a gradient of 0.06 μm^−1^. Interestingly, the y-intercept (*EF_HF_* (0)) is greater than 1, indicating that even at the tissue surface, there is an improvement. If no aberrations are present, both beam types are expected to give equal best performance at the tissue surface, since aberrations caused by the tissue will be minimal. This result is likely due to refractive index variations at the immersion medium/agarose interface above the tissue and the oblique angle at which the light-sheet intersects the tissue section. The analysis was repeated across images of different regions of the tissue and different tissue sections (sample size = 11).

As the improvement in image quality with ALSM may be linked to aberration resistance, optical clearing [21] was used to reduce the aberrations of the tissue to test this hypothesis. A solution of 47% TDE in 0.1M PBS (see Methods) was used both as clearing solution and imaging buffer for the same tissue sections that had been imaged before clearing. Images were acquired in similar regions to those imaged pre-clearing (sample size = 9).

Table 2 shows the average linear fit parameters (mean ± standard deviation) for *EF_HF_* (*z′*) in non-cleared and cleared tissue. There is an approximately five-fold reduction in the gradient of *EF_HF_* (*z′*) in cleared tissue. This confirms that the enhanced image quality of ALSM is linked to the aberration resistance of the Airy beam. The y-intercept is slightly higher in cleared tissue than in non-cleared tissue. We attribute this to additional aberrations caused at the microscope objective/imaging buffer interface, as the objective is not optimised for operation in media with the refractive index of the TDE solution (*n* = 1.42 ± 0.01 [21]).

**Table 2:** Linear fit parameters of *EF_HF_* (*z′*) in non-cleared and cleared mouse brain tissue. Sample size was 11 for non-cleared tissue, 9 for cleared tissue.

**Tissue Type**	**Gradient (**μ**m^−1^)**	**y-intercept**
Non-cleared	0.04 ± 0.02	1.2 ± 0.2
Cleared	0.008 ± 0.007	1.6 ± 0.4

**Fig. 4:**
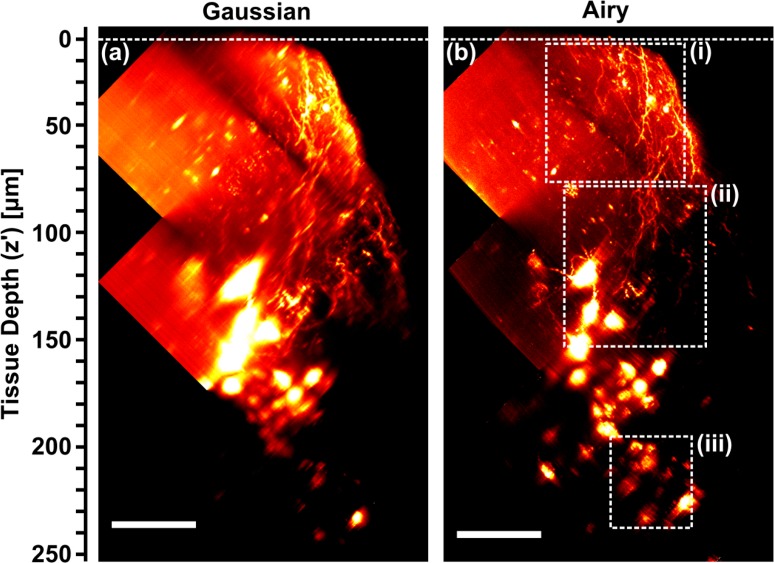
(a,b) Maximum intensity projections (*x′* − *z′* view) of fluorescent neurons in 400 μm thick, cleared mouse brain tissue section acquired using GLSM (a) and ALSM (b). Composite images of 3 datasets at different tissue depths. Dashed white lines indicate tissue surface. Cropped regions of (a,b) within dashed boxes (i–iii) indicate regions used for analysis in Fig. 5. Scale bar: 50 μm. This dataset can be accessed at [23].

Optical clearing also allows data to be collected from much deeper within the tissue section. Figure 4 shows composite images acquired by GLSM (a) and ALSM (b) at 3 different depths within the tissue. Key morphological features of mCherry-filled kisspeptin neurons were easier to recognize in the ALSM rendering, especially at greater tissue depth. Greater numbers of cell bodies and finer neuronal structures were evident, including neuronal dendrites (identified by their tapered shape extending from the cell body or the presence of spines) and axons (fine fibres with varicosities) throughout the brain section. Kisspeptin neurons in the hypothalamic arcuate nucleus are predicted to form recurrent collaterals (branched nerve output fibres looping back to the cell body) as well as form connections to the dendrites of neighbouring neurons in order to coordinate neurohormone release. Only the ALSM rendering displayed identifiable neuronal fibres between neighbouring kisspeptin cell bodies, indicating a clear advantage of ALSM for neuroanatomical studies.

**Fig. 5:**
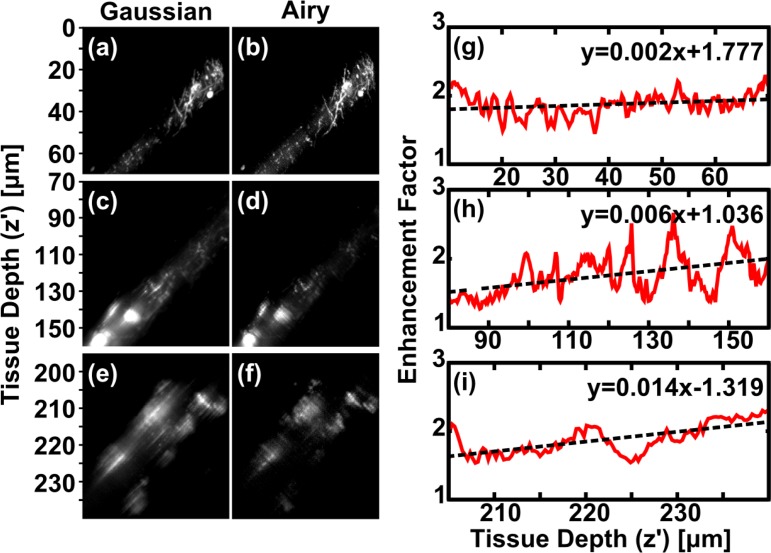
Fourier analysis of datasets shown in Fig. 4. (a–f) Cropped and apodized regions within dashed boxes (i–iii) from Fig. 4 acquired with GLSM (left) and ALSM (right). (g–i) High-frequency enhancement factor, *EF_HF_* (*z′*) within dashed boxes (i–iii). Dashed lines in (g–i) are linear fits to the data, equations shown on each plot. This dataset can be accessed at [23].

Regions of interest from Fig. 4 were apodized around Gaussian beam focus (Fig. 5(a–f)) and *EF_HF_* (*z′*) determined and fitted (Fig. 5(g–i)). The gradient of *EF_HF_* (*z′*) increases with increasing depth into tissue, and this behaviour was observed in 4 out of 5 composite, multi-depth datasets. This is to be expected, as aberrations are cumulative and should increase with depth. The variation in slope with depth also highlights that a simple linear model is not sufficient to capture the full complex behaviour through an entire tissue section, although over small subsections a linear model was appropriate in most cases we encountered.

### 3.2. Analysis of homogeneous features in bead-injected tissue

**Fig. 6:**
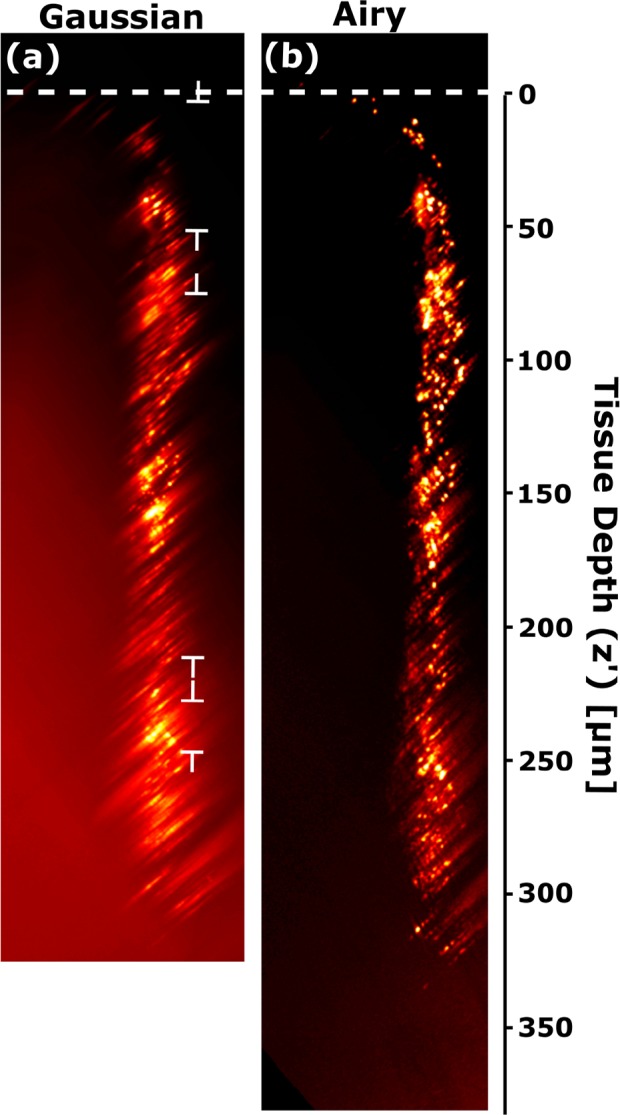
Maximum intensity projection (*x′* − *z′* view) of mouse brain tissue injected with red fluorescent microspheres (diameter 600 nm) and illuminated by (a) GLSM and (b) ALSM. The top of the tissue is indicated by a dashed white line. This dataset can be accessed at [23].

To control for the effects of inhomogeneous feature size and fluorescence intensity in the transduced tissue, we introduced a second sample. Fixed wild-type mouse brain tissue was injected with 600nm diameter red fluorescent polystyrene beads (polydispersity ±50 nm), which act as uniform resolution probes. Figure 6(a,b) show *x′* − *z′* projections of the image stacks acquired by Gaussian (a) and Airy (b) light-sheets in cleared tissue. The tissue was cut through the injection site and oriented such that the beads were in the side of tissue closest to the detection objective lens.

The enhanced uniformity and overall quality of the bead point-spread-function (PSF) in the Airy image compared to the Gaussian image are apparent to the eye. To quantify the difference, we used a spot-finding algorithm written in-house in MATLAB to identify PSFs within a designated size range (0.6 − 4.0 μm full width at half maximum) and fit 1D Gaussian functions along the lateral (*x*) and axial (*z*) directions. Table 3 summarizes the linear fits to the variation of the full widths at half maximum (FWHM) as a function of depth.

**Table 3:** Linear fits to the plot of FWHM vs depth from Figure 6. FWHM at surface (y-intercept) and FWHM/depth (gradient). Sample size (number of detected beads) was 86 for GLSM, 257 for ALSM.

**Beam Type**	**Fit Orientation**	**FWHM at surface (**μ**m)**	**FWHM**/**Depth (**μ**m**/ μ**m)**

Airy	Lateral	1.18 ± 0.04	(1.7 ± 0.2) × 10^−3^
	Axial	1.44 ± 0.06	(2.6 ± 0.3) × 10^−3^

Gaussian	Lateral	1.1 ± 0.1	(1.9 ± 0.6) × 10^−3^
	Axial	1.16 ± 0.09	(2.2 ± 0.6) × 10^−3^

For both beam types, the axial PSF increases more rapidly with depth than the lateral PSF. This is expected since the axial PSF is known to be the more sensitive of the two to aberrations in the excitation beam [28]. In addition, the beads are located at the edge of the tissue nearest to the detection objective lens, and aberrations in the optical pathway between fluorescence emission and detection are expected to be low compared to aberrations in the illumination pathway. Across beam types, the gradients of lateral and axial FWHM are the same to within fitting error, as are the lateral FWHM at the surface (y-intercepts). The only systematic difference is that the surface axial PSF with GLSM is smaller than with ALSM by approximately 0.3 μm.

This close numerical similarity fails to capture the qualitative difference between the Gaussian and Airy images. However, GLSM only performs so well in the numerical comparison because the spot-finding algorithm, which locates local maxima in the image and then evaluates the surrounding pixels to determine whether it has identified an approximately Gaussian PSF of acceptable size, automatically rejected much of the Gaussian image from its analysis due to blurring in the *x* − *z* plane. Accordingly, the analysis of FWHM vs depth oversells the quality of the Gaussian image by ignoring the large regions in Fig. 6(a) where the Gaussian light sheet is very broad and gives a large axial PSF. In these regions, the PSFs of individual beads are severely distorted, and their overlap exceeds the size range for the spot-finding algorithm. Close inspection of the figure reveals three regions of best focus, indicated by flat-ended arrows, corresponding to the center of the Gaussian light sheet for each of the three images in the stack. Meanwhile, the PSFs identified in the ALSM image are evenly distributed throughout the volume, including the regions where the GLSM image displays fluorescent features but no clear spots. It performs as well across the entire depth of the tissue as the Gaussian light sheet does in its optimal focus regions.

Finally, ALSM achieves higher depth penetration than GLSM. Although fluorescent features are distinguishable below 300 μm in the GLSM image, the deepest PSF whose FWHM and position could be evaluated was 251±1 μm below the surface of the tissue. Using ALSM, the size and position of beads at a depth of 330±1 μm, very close to the bottom of the 400 μm slice, could still be resolved. Use of ALSM increased depth penetration into this tissue by approximately 30% (80 μm).

## 4. Summary and conclusions

We have used three methods to assess the effect of illumination beam shape on the imaging performance of LSM in mouse brain tissue. To compare Gaussian- and Airy-LSM, we developed an image quality metric based on the magnitude of the spatial Fourier transform of an image within a certain spectral window. This method is general and can be applied across a wide range of imaging techniques to compare image quality on a common sample. The method is also robust to single-pixel noise fluctuations, as these typically occur at spatial frequencies which are rejected by the analysis in an appropriately sampled image. A depth-dependent enhancement factor was observed, and a linear model (*EF*_*HF;non*–*cleared*_ (*z′*) = 0.04*z′* + 1.2) fitted this well. This model indicates an average enhancement of 3.2 times at a depth of 50 μm within non-cleared mouse brain tissue.

Additionally, optical clearing was used to control the aberrations caused by the sample. Clearing greatly reduced the sample-induced aberrations, the average enhancement factor for surface level datasets was reduced compared to in non-cleared tissue, and could be described by a linear model (*EF_HF;cleared_* (*z′*) = 0.008*z′* + 1.6). The gradient of the linear model in cleared tissue is 5 times less than in non-cleared tissue. This model indicates an average enhancement factor of 2 times at a depth of 50 μm within cleared tissue. Comparing images of cleared tissue at different depths revealed an increase in enhancement factor with increasing depth, or increasing cumulative aberration. Both these results indicate that the enhancement factor is linked to the degree of sample-induced aberration, and that the improvement observed with Airy-LSM is related to the aberration resistance of the Airy beam.

Finally, the maximum achievable imaging depth was investigated for each beam type using the same brain tissue injected with resolution probes. Both Gaussian-LSM and Airy-LSM gave similar results for lateral and axial resolution but Airy-LSM enabled imaging 30% (80 μm) deeper into the tissue than Gaussian-LSM.

The source of the enhancements observed with Airy-LSM may be attributed to an inherent aberration resistance of the Airy beam profile. This natural resistance to aberrations can be linked to the self-healing phenomenon which has been previously reported for Airy beams [14–19]. The parabolic caustic of maximum intensity, which is the main feature of the Airy beam, can be visualised from a ray optics perspective [16]. In this picture, different rays, each corresponding to different k-vectors, contribute to the caustic shape at different points along the propagation axis. As such, each longitudinal section of the beam samples a different region of the pupil. Aberrations that affect one point of the beam will have a lesser effect on other regions of the beam profile [16] and the effects of aberrations on a small sub-section of the pupil may be much less severe than on the pupil as a whole [29]. A more rigorous wave optics treatment of Airy beam formation has suggested that the robustness of the beam is directly linked to the *π*–phase shifts between adjacent lobes of the Airy profile [15]. Bessel beams have similar phase structure [30], which has also been shown to resist the effects of aberration in turbid media [13]. Our work presents robust data for the first time that the Airy beam shows resistance to tissue aberrations and as such, holds promise for imaging.

Extracting high-quality volumetric information from within complex tissue is an immediate challenge in neurobiology. For instance, the kisspeptin neurons labelled here are integral to several complex neuronal circuits forming intimate connections with neighbouring neurons as well as long distance connections traversing the brain. The availability of enhanced imaging techniques to reveal these types of elaborate morphological features would greatly contribute to systems neuroscience. Our analysis has shown that Airy-LSM generates superior image quality in both cleared and non-cleared neural tissue over Gaussian-LSM due to the natural aberration resistance of the Airy beam. Recent innovations in optical clearing methods [21, 24–26] are rapidly advancing large-scale 3-dimensional neural connectivity studies [27]. Through this study, we have shown that Airy-LSM is compatible with one such optical clearing method (TDE [21]), making it immediately applicable to these areas, especially as the technique can be implemented in a compact and inexpensive manner [10] that is very suitable for end-users.
